# Immunity through Leucomaines

**Published:** 1889-08

**Authors:** 


**Immunity through Leucomaines.** By EUSEBIO GUELL BACIGALUPI. Translated from the second French edition by R. F. RAFAEL, M. D. New York: J. H. Vail & Co. 1889.

The author divides his treatise of 155 pages into three parts. In the first part he discusses his theory of immunity ; in the second part follows the demonstration of the theory ; and in the third part he explains some of the phenomena of infectious diseases by this theory.

In Part I., after discussing briefly the various existent theories on vaccination and immunity, he finds the explanation of the various phenomena produced by microbes as very unlikely. The starting-point which led him to a new path of reasoning was taken from a note of Mr. Ferran:

> The microbe does not reproduce in the cellular tissue, and its prophylactic action is due to a sort of accustoming or habituation of the organism to the active diffusible substance generated by the microbe.

It is not, then, the microbe more or less attenuated which produces immunity from a disease, but the substance generated by the microbe.

The author supports his thesis by several modes of reasoning, showing that in living organisms, whether animal or vegetable, the products of life are noxious to this same life. Applying this principle to the microbe, the excretory matter of the microbe, or leucomaines, renders its life feeble and finally causes its death.

The conclusions to be drawn are in keeping with this same fact. The state of immunity in an individual, whether having previously suffered from an infectious disease, or one in whom leucomaines have been artificially introduced, depends upon the leucomaines still present in the body.

Vaccination, to preserve from infectious disease, should, therefore, consist in introducing into the organism the leucomaines of the microbe that produces the disease against which protection is sought.

In Part II. follows the analyses of some of the infectious diseases in which vaccination has been attempted and proven successful. In fowl cholera, in anthrax, and in rabies, it is not the vaccine of the specific microbe in an attenuated form which shields from the disease, but the leucomaines of these microörganisms. Especially in the prophylaxis of rabies the author dwells at some length, and in a clear and forcible manner shows that the leucomaines must be considered as the prophylactic agent.

In the succeeding chapters, the writer treats of the nature of inherited immunity, considers the various methods of vaccination, the attenuation of virus, etc.

Part III. is devoted to the explanation of some of the phenomena observed in the course of infectious diseases. Accepting the theory of leucomaines, the explanations offered appear quite plausible, and help to clear up some very intricate points. W. C. K.

